# Expression of Desmocollin-1 in Carotid Atherosclerotic Plaques and Calcific Aortic Valves

**DOI:** 10.1159/jvr/adiag006

**Published:** 2026-10-09

**Authors:** Kashif Khan, Bin Yu, Sarah Abdulkarim, Adam Schwertani, Jacques Genest, Stella S. Daskalopoulou, Adel Schwertani

**Affiliations:** a Division of Cardiology, Department of Medicine McGill University Health Center, Montreal, QC, Canada; b Division of General Internal Medicine, Department of Medicine McGill University Health Center, Montreal, QC, Canada

**Keywords:** Desmocollin-1, Carotid plaque, Atherosclerosis, Aortic valve, Human, Immunohistochemistry, RT-PCR, Smooth muscle cells, Macrophages

## Abstract

**Introduction:**

Atherosclerosis and calcific aortic valve stenosis are active, regulated processes sharing features, such as lipid infiltration, extracellular matrix deposition, cellular proliferation, and calcification. Their exact mechanisms remain unclear. High-density lipoprotein (HDL) is central to reverse cholesterol transport, and Desmocollin-1 (DSC1) has recently been implicated in HDL biogenesis. This study assessed DSC1 expression and localization in atherosclerotic plaques and calcific aortic valves.

**Methods:**

Human carotid artery specimens (19 subjects: plaques and normal tissue) and aortic valves (50 subjects: stenotic calcific and normal) were collected. DSC1 mRNA expression was measured by quantitative RT-PCR, and protein expression was evaluated using immunohistochemistry and Western blot analyses.

**Results:**

DSC1 mRNA and protein were abundantly expressed in carotid plaques and stenotic calcific valves, but not in normal tissues. In calcified valves, DSC1 localized to lipid cores, inflammatory cells, and microvessels. In carotid plaques, DSC1 was mainly found in foam cells and myointimal cells.

**Conclusion:**

DSC1 expression is significantly increased in both atherosclerotic plaques and stenotic calcific valves, suggesting a role in lipid metabolism abnormalities. These findings point to DSC1 as a potential contributor to disease pathogenesis and warrant further targeted investigation.

## Introduction

Much of our knowledge of calcific aortic valve stenosis (CAVS) comes from atherosclerosis, as they both have similar pathophysiology [1]. Both diseases begin to manifest after excess lipid deposition in the wall of the artery or aortic valve, resulting in the formation of fatty streaks [2]. The process of cellular proliferation and increased matrix deposition ensues, which progresses to inflammation, calcification, and new vessel formation with increased severity in both diseases [3]. Given the similarities between atherosclerosis and CAVS, a therapy that targets and reverses lipid accumulation that initiates their pathogenesis would be highly beneficial.

Dysregulated lipid biomarkers, such as increased total cholesterol, low-density lipoprotein cholesterol (LDL-C), and low levels of high-density lipoprotein cholesterol (HDL-C), are commonly noted in carotid atherosclerosis. One of the hallmarks of atherosclerosis is that excess LDL-C promotes lipid accumulation within the arterial intima, undergoes oxidative modification, accumulates in macrophages, and leads to foam cell formation and the development of lipid-rich plaques [2]. However, the role of these biomarkers in CAVS remains controversial. Some studies have indicated an association of circulating total cholesterol and LDL-C with CAVS progression [4, 5], while other studies show no association [6]. Furthermore, emerging evidence highlights a causal relationship of lipoprotein(a) [Lp(a)] levels with CAVS, suggesting that elevated Lp(a) contributes to valvular calcification due to its pro-inflammatory and pro-oxidative properties [7, 8]. Large genetic and population studies have reinforced this view, demonstrating that variants leading to higher Lp(a) concentrations are significantly associated with an increased risk of CAVS, independent of other lipid profiles [9–11]. In contrast, several studies suggest a lack of association between HDL-C and CAVS progression, largely limited by low sample sizes [12–14]. In other studies, HDL-C was found to be lower in patients with CAVS [6] and implicated in aortic valve calcium deposition [15]. Furthermore, total cholesterol/HDL-C ratio has been associated with CAVS progression, while HDL-C alone was inversely associated with hemodynamic progression of the disease [16]. Serum HDL-C is associated with macrophage cholesterol efflux capacity, which is a strong predictor of atherosclerosis status [17]. Impaired macrophage cholesterol efflux promotes lipid retention within foam cells and the development of lipid-rich necrotic cores, a key feature associated with plaque progression and instability. Although further research is needed, these findings suggest that HDL-related pathologies may be associated with the pathogenesis of both atherosclerosis and CAVS.

Desmocollin-1 (DSC1) has been recently identified to play an important role in the regulation of HDL-C biogenesis and reverse cholesterol transport in vitro [18, 19]. It acts by binding directly to circulating apoA-1, preventing the apoA-1 interaction with the ATP-binding cassette transporter A1 (ABCA1), the rate-limiting step in reverse cholesterol transport. Inhibition of the ABCA1-apoA-1 interaction prevents HDL biogenesis and removal of cholesterol from the cell. DSC1 also sequesters cholesterol on DSC1-containing plasma membrane microdomains, further inhibiting the release of cholesterol from the cell [20]. The DSC1 gene encodes DSC1A and DSC1B, which differ in their -COOH cytoplasmic domain where DSC1A has a larger variant of 65 amino acids and DSC1B has a smaller 11 amino acid variant [21]. DSC1A is critical for assembling the desmosomal plaque connecting desmosomes to the intermediate filament cytoskeleton in epithelial cells [21]. DSC1A uniquely binds to plakoglobin and desmoplakin for plaque assembly. DSC1B is also integrated in desmosomes, and in the context of atherogenesis has been shown to increase apoA-I binding capacity [22, 23]. Thus, the targeting of DSC1 subtypes may provide more information on HDL handling and provide additional lipid-lowering benefits. In the present study, we examined the expression of DSC1 in diseased tissue (carotid plaques and stenotic calcified aortic valves) in comparison to normal tissues.

## Methods

### Tissue Collection

Carotid artery specimens were obtained from 19 subjects (16 with atherosclerotic plaques [*n* = 5 for RT-PCR, *n* = 6 for Western blot, *n* = 10 for IHC], 3 normal), and aortic valves from an additional 50 subjects (45 diseased [*n* = 31 for RT-PCR, *n* = 12 for Western blot, *n* = 10 for IHC], 5 normal). The cardiovascular profile of patients with carotid artery disease and CAVS is described in Tables 1 and 2, respectively. Carotid atherosclerotic plaque specimens were obtained from carotid endarterectomies, while normal carotid tissues from organ transplant donors. Diseased and normal aortic valves were obtained from valve replacement surgeries and heart transplant donors, respectively. Tissues were freshly collected during surgery; part of the tissue was fixed in formalin and embedded in paraffin for immunohistochemical analyses, and the remaining piece was snap frozen in liquid nitrogen and stored at −80°C until processed for quantitative real-time reverse transcription polymerase chain reaction (qRT-PCR) and Western blot analyses. The study was approved by the Ethics Committee of the McGill University Health Centre, and informed consent was obtained from all participants involved in the study.

**Table 1.** Clinical characteristics of the carotid artery disease patients included in quantitative real-time reverse transcription polymerase chain reaction, Western blot, and immunohistochemistry studies

Clinical parameter	qRT-PCR and Western blot	IHC
	mean±SD (*N* = 6)	mean±SD (*N* = 10)
Age, years	78.7±9.1	65.5±7.7
Female, *n* (%)	2 (33)	3 (30)
Diabetes, *n* (%)	1 (17)	2 (20)
Total cholesterol (mmol/L)	2.7±0.5	4.7±1.8
LDL-C (mmol/L)	1.0±0.6	2.9±1.6
HDL-C (mmol/L)	0.8±0.2	0.9±0.2
Triglycerides (mmol/L)	1.7±0.9	1.9±1.0

qRT-PCR, quantitative real-time reverse transcription polymerase chain reaction; SD, standard deviation; IHC, immunohistochemistry; LDL, low-density lipoprotein cholesterol; HDL, high-density lipoprotein cholesterol.

**Table 2.** Clinical characteristics of the aortic valve stenosis patients included in quantitative real-time reverse transcription polymerase chain reaction, Western blot, and immunohistochemistry studies

Clinical parameter	qRT-PCR	Western Blot	IHC
	mean±SD (*N* = 31)	mean±SD (*N* = 12)	mean±SD (*N* = 10)
Age, years	69.1±11.0	68.4±13.3	70.6±7.2
Female, *n* (%)	10 (32)	4 (33)	4 (40)
Diabetes, *n* (%)	5 (16)	3 (25)	3 (30)
Total cholesterol (mmol/L)	4.5±1.7	4.1±1.1	3.2±0.2
LDL-C (mmol/L)	2.9±2.0	2.6±1.0	1.5±0.03
HDL-C (mmol/L)	1.8±0.5	1.0±0.1	0.9±0.2
Triglycerides (mmol/L)	1.1±0.3	1.0±0.1	1.6±0.7
Aortic valve area (cm^2^)	1.5±0.7	1.0±0.4	1.0±0.4
Jet velocity, cm/s	293±117	370±186	366±187
Pmax, mm Hg	39.3±31.9	65.1±52.9	63.0±48.0
Pmean, mm Hg	24.7±22.3	40.3±33.2	39.5±30.8
Ejection fraction (%)	55.9±14.4	57.5±11.9	53.3±11.5

qRT-PCR, quantitative real-time reverse transcription polymerase chain reaction; SD, standard deviation; IHC, immunohistochemistry; LDL, low-density lipoprotein cholesterol; HDL, high-density lipoprotein cholesterol.

### Gene Expression Analysis

Total RNAs were extracted from snap frozen carotid arteries and aortic valve tissues using TRIzol (Invitrogen)/RNeasy Mini Kit (QIAGEN) combined protocol. Total RNA (1 μg) was used for first-strand cDNAs synthesis using SuperScript III First-Strand Synthesis System (Invitrogen). qRT-PCRs were performed using QuantiFast SYBR Green PCR kit (QIAGEN) on LightCycler 1.5 (Roche). The qRT-PCR data were analyzed using PrimePCR Analysis (Bio-Rad), and the relative mRNA expressions of the genes of interest were normalized using the housekeeping gene GAPDH. Primer sequences are described in online supplementary Table 1 (for all online suppl. material, see https://doi.org/10.1159/jvr/adiag006).

### Western Blot Analyses

Tissues or cells were lysed and re-suspended in RIPA buffer supplemented with complete™, Mini, EDTA-free Protease Inhibitor Cocktail (Sigma-Aldrich) for protein extraction. Samples were then centrifuged at 15,000 rpm at 4°C for 10 min and supernatants were collected, protein concentrations were measured by Protein DC protocol (Bio-Rad), and equal amounts were loaded for 10% SDS-PAGE gel separation. The protein was transferred onto PVDF membrane at 300 mA for 1 h at room temperature, blocked for 1 h at room temperature with 5% milk (1xTBST buffer), then incubated with primary antibodies against DSC1 and their dilutions summarized in online supplementary Table 2 for 16 h at 4°C; then followed by incubation with anti-rabbit IgG-HRP (sc-2371, 1:3000). For signal development, Lumi-Light Western blot Substrate (Sigma-Aldrich) was used according to manufacturer protocols.

We tested and optimized four different antibodies against DSC1 and its two variants (DSC1A and DSC1B) (Fig. 1). The remainder of this study directly compared the protein expression by Western blot of the Ab150382 antibody and the antibody developed by our lab (Lab).

**Fig. 1.**
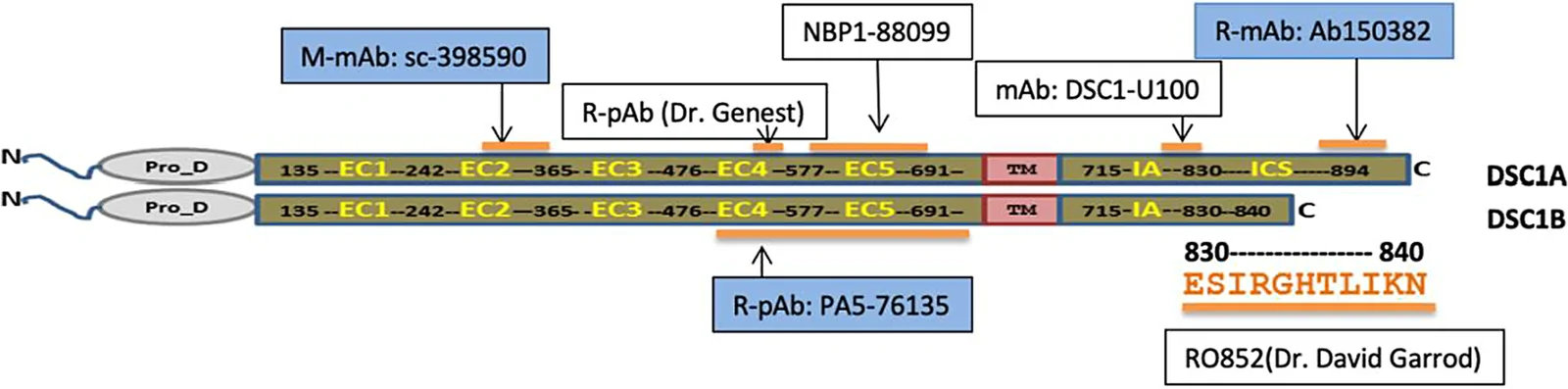
Protein sequence schematic of Desmocollin-1 (DSC1)A and DSC1B. Known antibodies and their reaction domains. The four antibodies tested and optimized in this study are further described in online supplementary Table 2. Antibodies tested in this study are highlighted in blue. EC = Extracellular domain used in; IA = intracellular anchor; ICS = intracellular cadherin-like sequence; TM = Transmembrane helix.

### Immunohistochemistry

The paraffin-embedded tissue blocks were cut into 4 μm sections using a microtome. The sections were incubated for 1 h in 10% normal goat serum/PBS solution, then incubated overnight with the primary antibodies in 0.1% BSA/PBS solution in humid chambers at 4°C [24–27]. Primary antibodies used are shown in online supplementary Table 2, which includes the use of our own antiserum, as previously described [18]. Secondary antibody Mouse/Rabbit IgG VisUCyte HRP Polymer (R&D, # VC002-025) were applied followed by Vectastain ABC complex (Vector Laboratories, Burlingame, CA, USA) according to manufacturer protocol. Immunostaining was visualized by 1×DAB/H_2_O_2_ solution, subsequently counterstained with hematoxylin, and mounted with Permount (Sigma-Aldrich). Negative control sections were prepared either by omitting the primary antibody or by using primary antiserum preabsorbed with the corresponding DSC1 antigen. Positive control immunostaining of DSC1 was performed on skin and myocardial tissue (online suppl. Fig. S1a and b, respectively). Immunoreactivity was semiquantitatively evaluated using the immunoreactive score method [28] which scores staining intensity (0–3) multiplied by the percentage of positive cells (0–4).

### Statistical Analysis

Unpaired Student’s *t* tests were used to assess statistically significant differences in mRNA and protein expression measurements of DSC1 between diseased and normal control tissue samples. Statistical analysis was performed using SPSS program Version 15. Data are presented as mean ± standard deviation.

## Results

### DSC1 and Carotid Atherosclerosis

DSC1 mRNA analysis revealed significantly increased expression in carotid plaques compared to healthy control samples (*p* < 0.05; Fig. 2a). Western blot analyses revealed no expression of DSC1A protein in normal carotid artery tissues when using Ab150382, while carotid plaques showed clear expression of the DSC1 protein (*p* < 0.001; Fig. 2b, c). Similar results were obtained when utilizing our lab’s antibody against both DSC1A and DSC1B (*p* < 0.05; Fig. 2d, e). Both forms of the DSC1 protein were clearly expressed in diseased vessels compared to no signal in normal vessels.

**Fig. 2.**
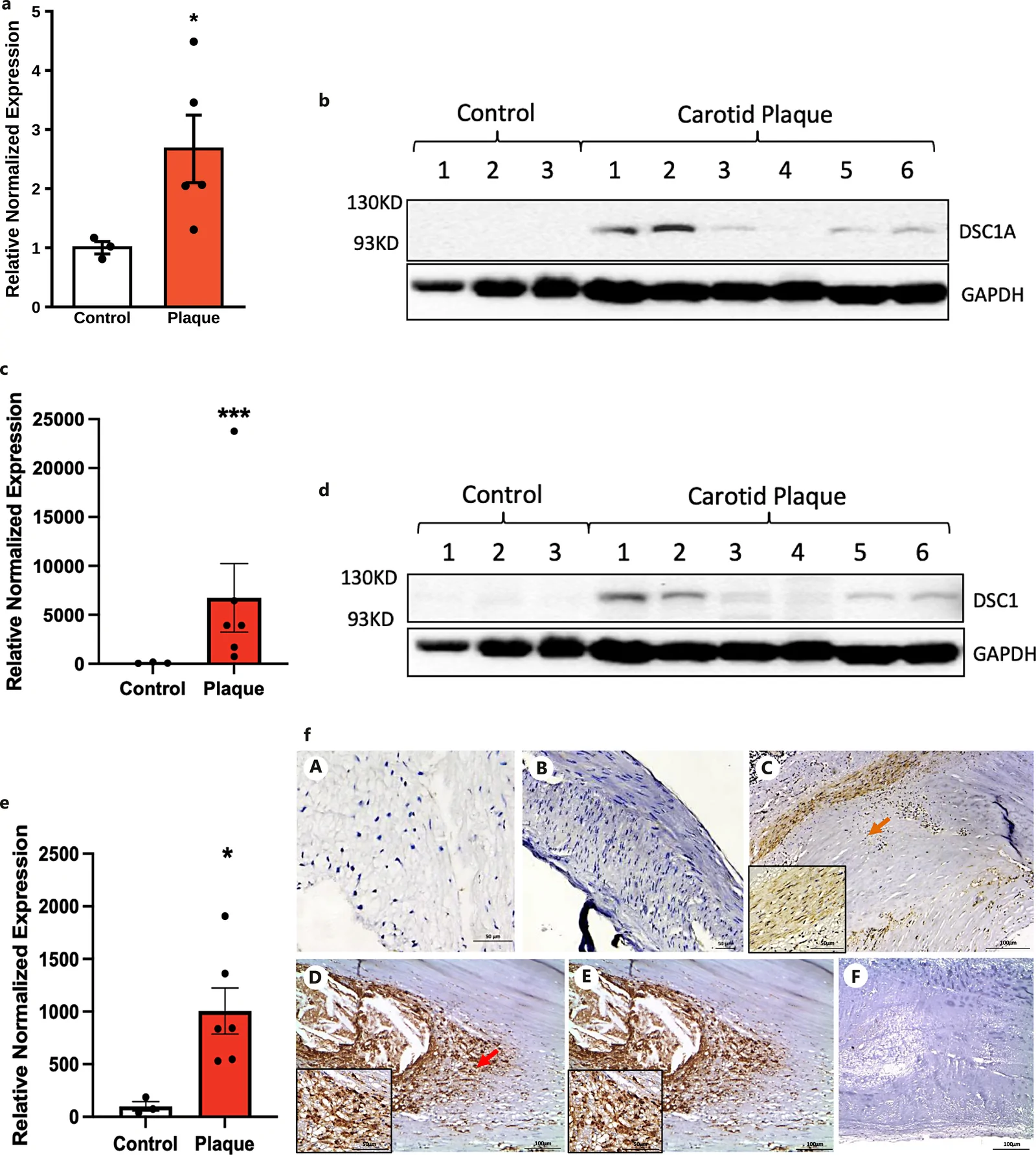
Desmocollin-1 (DSC1) expression in carotid artery disease. mRNA expression analysis of DSC1 in carotid plaques (*n* = 3 for control, *n* = 5 for carotid plaques) (**a**). Representative Western blot analyses of DSC1 protein expression in carotid plaques using Ab150382 (**b**) and the respective relative normalized quantifications of the blot (*n* = 3 for control, *n* = 6 for carotid plaques) (**c**). Representative Western blot analyses of DSC1 protein expression in carotid plaques using the Lab’s antibody for DSC1A and B (**d**) and the respective relative normalized quantifications of the blot (*n* = 3 for control, *n* = 6 for carotid plaques) (**e**). Representative immunohistochemical images of DSC1 protein expression in foam cells and myointimal cells in carotid plaques (**f**): negative control using primary antiserum preabsorbed with the DSC1 antigen (**f–**A), no DSC1 immunostaining in normal carotid arteries (**f–**B), DSC1 immunostaining in areas of fibrosis (**f–**C), DSC1 immunostaining in the lipid core (**f–**D), serial immunostaining of CD68 in the lipid core (**f–**E), hematoxylin counterstain in absence of the primary antisera in a carotid artery (**f–**F). Red arrows represent macrophages and foam cells around the lipid core. Orange arrows represent areas of fibrosis.

In terms of immunohistochemistry experiments, negative control sections immunostained with antisera preabsorbed with the DSC1 antigen showed no immunoreactivity (Fig. 2f-A). There was no apparent immunoreactivity for DSC1 in normal carotid arteries (Fig. 2f-B). Conversely, regions exhibiting atheromatous changes demonstrated a pronounced immunoreactivity for DSC1. DSC1 immunostaining was seen in myointimal cells (Fig. 2f-C, orange arrows). The localization of DSC1 was particularly striking in areas characterized by early macrophage infiltration and foam cells (Fig. 2f-D and Fig. 2f-E), red arrows). Representative tissue section stained with hematoxylin alone, in absence of the primary antisera, is shown in Figure 2f-F to demonstrate tissue morphology and cellular architecture. Positive control sections of human myocardial tissue and skin showed strong immunoreactivity for DSC1 (online suppl. Fig. 1).

### DSC1 and Aortic Valve Stenosis

qRT-PCR revealed increased expression of DSC1 mRNA in stenotic calcific aortic valves compared to normal control samples (*p* < 0.01; Fig. 3a). Western blot experiments showed faint and varied expression of DSC1A (Ab150382) in calcific aortic valves (Fig. 3b). Using the lab antibody, there was abundant expression of DSC1A and DSC1B in calcific aortic valves, with minimal expression in control tissues (*p* < 0.01; Fig. 3c, d).

**Fig. 3.**
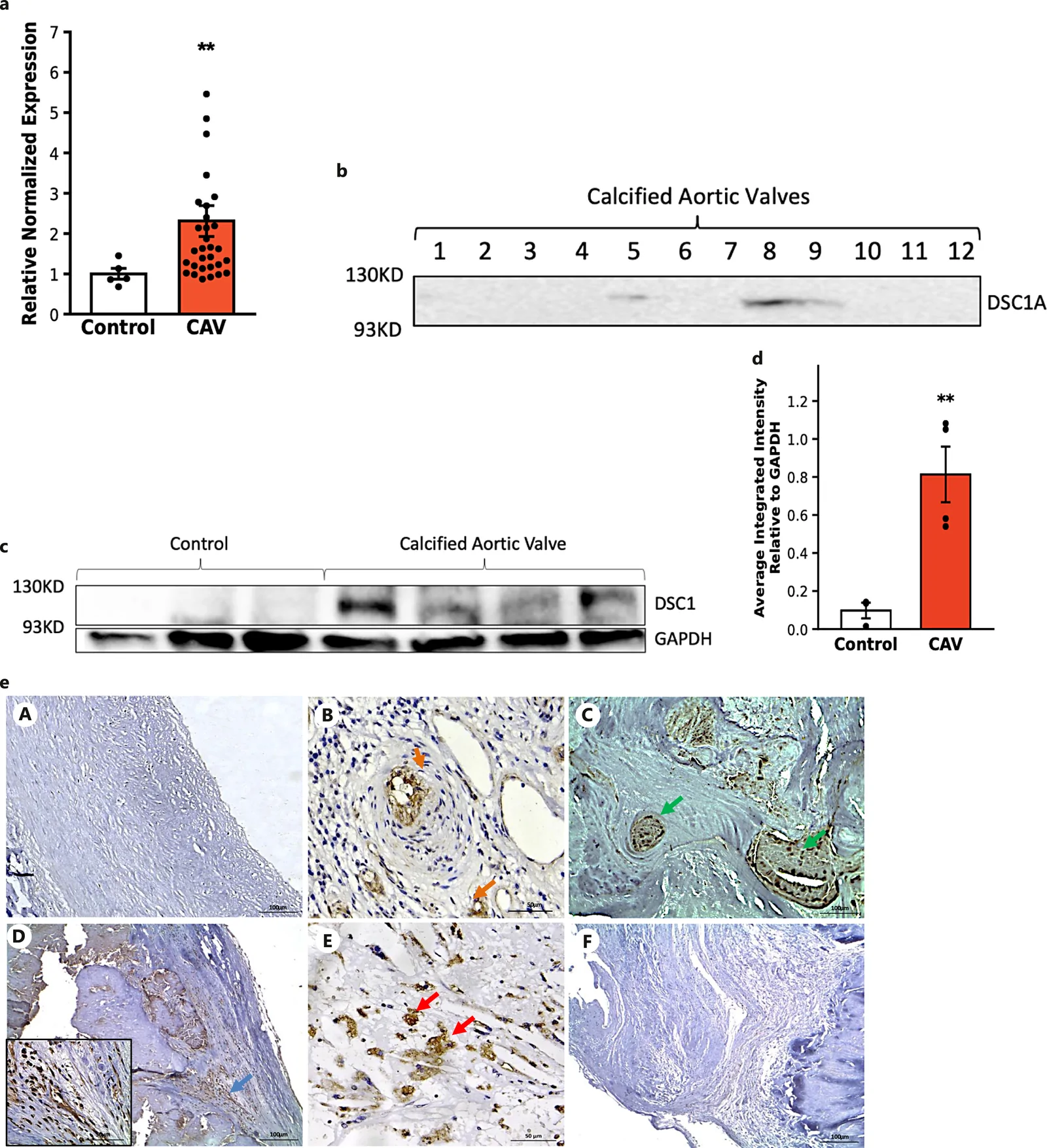
Desmocollin-1 (DSC1) expression in CAVS. **a** mRNA expression analysis of DSC1 in calcified aortic valves (*n* = 5 for control, *n* = 31 for calcified aortic valves). **b** Representative Western blot analyses of DSC1 protein expression in calcified aortic valves using Ab150382 (DSC1A) (*n* = 12 for calcified aortic valves). **c** Representative Western blot analyses of DSC1 protein expression in calcified aortic valves using the Lab’s antibody (DSC1A and B). **d** And the respective relative normalized quantifications of the blot (*n* = 3 for control, *n* = 4 for calcified aortic valves). **e** Representative immunohistochemical images of DSC1 protein expression in the atheroma and in areas of fibrosis, calcification, neovascularization, and foam cell formation: no DSC1 immunostaining in normal aortic valves (**e–**A), DSC1 immunostaining around microvessels (**e–**B), DSC1 immunostaining in fibrotic core with areas of calcification (**e–**C), DSC1 immunostaining in areas of inflammation around fibrotic cores (**e–**D), DSC1 immunostaining in tissue macrophages (**e–**E), hematoxylin counterstained negative control processed in the absence of the primary antibody in an aortic valve (**e–**F). Red arrows represent macrophages and foam cells. Orange arrows represent endothelial cells around microvessels. Green arrows represent calcification nodules.

There was no significant immunoreactivity for DSC1 in normal sections of aortic valves (Fig. 3e-A), nor in hematoxylin stained sections in absence of the primary antisera (Fig. 3e-F). In contrast, strong DSC1 immunoreactivity was observed in calcific aortic valves. Specifically, DSC1 immunostaining was seen in endothelial cells in areas of microvessel formation (Fig. 3e-B, orange arrows), fibrotic cores with calcified regions (Fig. 3e-C) and areas of inflammation around the fibrotic cores (Fig. 3e-D, blue arrows). DSC1 immunostaining was also prominent in macrophages and foam cells (Fig. 3e-E, red arrows). Representative tissue section stained with hematoxylin only is shown in Figure 3e-F, demonstrating tissue morphology and cellular architecture.

### Semiquantitative Immunoreactivity Scoring

Semiquantitative immunoreactive scoring (IRS; range 0–12) demonstrated minimal to absent DSC1 expression in control carotid specimens, with most non-atherosclerotic sections showing no detectable immunoreactivity (IRS = 0). In contrast, carotid atherosclerotic lesions exhibited marked DSC1 expression, with strong immunoreactivity observed within atheromatous regions, particularly in intimal smooth muscle cells, macrophages, foam cells, monocytes, and endothelial cells, frequently reaching maximal IRS values (IRS = 12). Early lesions displayed lower but detectable staining, primarily localized to macrophage-rich areas (IRS = 4). Similarly, CAVS specimens showed robust DSC1 expression in severe disease, with multiple cases demonstrating strong and widespread immunoreactivity (IRS = 12), while milder or fibrotic lesions exhibited focal or reduced staining (IRS = 3–6). Overall, DSC1 expression increased with lesion severity and inflammatory burden across both carotid and CAVS samples.

## Discussion

Atherosclerosis and CAVS fundamentally share similar pathogenesis [3, 29]. They both are inflammatory processes that begin with endothelial dysfunction, triggering intimal thickening, smooth muscle cell proliferation and migration [2]. Macrophage infiltration and the development of foam cells is a hallmark of both diseases, leading to plaque instability and rupture in atherosclerosis and valvular dysfunction in CAVS [30–32]. The local inflammatory environment also induces osteoblast differentiation of local progenitor cells, leading to significant calcification within the vessel and valve [1, 3, 33]. Our lab previously showed that DSC1 is abundantly expressed in areas of lipid core, cholesterol crystal deposition, and calcification in coronary plaques and in tissue macrophages [18]. The present study extends this evidence and demonstrates that DSC1 is highly expressed in carotid plaques and calcified aortic valves, but not in normal tissue, supporting the need for further investigation into DSC1’s role in atherogenesis.

We tested DSC1 gene expression using several antibodies and compared one which binds specifically to DSC1A, while the other binds to regions in both DSC1A and DSC1B. Indeed, the DSC1 gene encodes two proteins: DSC1A and DSC1B, which are largely homologous and only different in their COOH-terminal cytoplasmic amino acid sequences [21]. These variants are generated through differential splicing of the mRNA [34]. The DSC1A variant has more extended COOH-terminal sequences with 65 amino acids, while DSC1B concludes with a shorter 11 amino acid sequence [35]. Our testing of antibodies against both DSC1A and DSC1B allows comparison of the relative expression of these variants in diseased tissues. In our carotid atherosclerosis Western blot analyses, both the DSC1A and DSC1A/B antibodies showed abundant protein expression. In CAVS, however, the antibody targeting DSC1A showed faint expression in diseased valves, while the in-house antibody against both DSC1A and DSC1B showed clear expression in calcified aortic valves compared to normal ones, indicating similar or possibly greater DSC1B expression in calcified aortic valves. The current understanding of the *DSC1* gene suggests that its variants, DSC1A and DSC1B, may activate distinct signaling pathways such as Wnt-signaling, potentially contributing differently to the development of atherosclerosis and CAVS. As this is an emerging field of study, further research is essential to elucidate these pathways.

IHC studies revealed significant protein expression of DSC1A in areas of lipid and macrophage infiltration in both carotid atherosclerosis and CAVS. Differential protein expression between carotid atherosclerosis plaques and normal arteries was confirmed by Western blot analyses for the DSC1A antibody and the in-house DSC1A/B antibody. When a commercial antibody for DSC1A was used in twelve calcified aortic valves, there was faint or no expression in many of the tissues. However, the in-house antibody directed against both DSC1A and DSC1B showed abundant protein expression in Western blot analyses, while minimal expression was observed in normal aortic valves. Together, these results further demonstrate local DSC1 expression in atherosclerotic lesions, and differential expression of DSC1A and DSC1B in calcific aortic valves.

HDL particles play an integral role in reverse cholesterol transport, as well as other anti-atherosclerotic processes such as reduced inflammation, anti-thrombosis, and preventing monocyte attachment [19, 36]. Although increased HDL-C levels have been associated with decreased cardiovascular risk, clinical trials have shown that actively raising HDL-C levels does not induce cardioprotection [37–39]. Therefore, it is more likely that enhancing the function of HDL will provide anti-atherosclerotic benefits. One method to increase HDL biogenesis is to enhance the activity of ApoA-I. Our group has previously shown that DSC1, an apoA-I binding protein, is highly expressed around the lipid core and cholesterol crystals of atherosclerotic plaques [18]. Importantly, we showed that DSC1 blocks the interaction between ApoA-I and ABCA1, preventing the formation of HDL particles and the cholesterol efflux process. In the current study, we provide further insights into this process by showing increased expression of DSC1A in carotid plaques and calcified aortic valves, which highlights the potential importance of targeting this specific isoform when developing therapeutic targets. Reverse cholesterol transport is an essential mechanism in both atherosclerosis and CAVS pathogenesis, suggesting a potential association between DSC1 expression and lipid-handling pathways in diseased tissues.

Beyond their structural role, recent research has uncovered that desmosomal cadherins are also involved in signaling pathways that can mediate cellular processes like apoptosis [40]. Increased DSC1 expression is found in cancers due to activation of proliferation pathways [41]. When inhibited, RNA sequencing studies show decreased enrichment of proliferative pathways. In a rabbit model of atherosclerosis, Docetaxel-treated animals showed a 40% reduction in the proliferative cell nuclear antigen marker [42]. This was associated with reduced expression of several pro-apoptotic proteins including caspase 3, caspase 9, and Bax, along with no significant toxic side effects. Tissue macrophages are often converted to foam cells through uptake of oxidized lipoproteins coupled with the local pro-inflammatory environment, triggering the activation of apoptosis [43]. The release of the lipids, inflammatory cytokines, and cellular debris within the plaque promotes necrotic core formation, intraplaque hemorrhage, and subsequently plaque rupture [43–45]. Together, these observations suggest that DSC1-related pathways may contribute to processes involved in atherogenesis, such as lipid handling, inflammation, and plaque instability.

Given the potential systemic effects of modulating DSC1 signaling, therapeutic strategies targeting this pathway would likely benefit from approaches that enable localized delivery to diseased vascular tissues. Experimental studies using nanoparticle-mediated delivery of Docetaxel to atherosclerotic plaques in cholesterol-fed rabbits showed an 80% reduction in atheroma formation from the aortas compared to controls [42]. There was also a significant reduction in macrophages and inflammatory markers without evidence of hematological, hepatic or renal toxicity. In a more recent study, low-dose Docetaxel was given to ApoE−/− mice fed a high-fat diet for 2 weeks, achieving therapeutic drug levels, reduced size of atherosclerotic lesions in the aortic sinus and arch, and no evidence of hematological toxicity [46]. Interestingly, there were significant reductions in circulating atherogenic lipids, while increasing the HDL cholesterol/total cholesterol ratio. These findings support the feasibility of targeting DSC1-related pathways in vivo with early evidence suggesting minimal off-targeted effects, which need to be validated in larger animal models and humans.

One limitation of our study is that this is a cross-sectional study with descriptive analysis of tissue samples taken at a specific time. While our study draws strong correlation between DSC1 expression and the progression of CAVS and carotid atherosclerosis, it does not provide data to support a cause-and-effect relationship and additional studies that address this are warranted. Additionally, it is important to recognize that medication histories for the patients included in this study and clinical characteristics of the control group (i.e., donors) were not available and therefore could not be included in the analysis. These data were not accessible due to ethical and data access restrictions associated with donor anonymity. Furthermore, as the patients included had end-stage disease, many of them were likely on statins or anti-inflammatory agents. which may influence vascular biology and biomarker expression and could therefore represent potential confounders.

This study adds to the accumulating evidence for the role of DSC1 in atherogenesis. We describe the prevalence and cellular localization of DSC1A and total DSC1 in atherosclerosis and CAVS, namely, in lipid-rich plaques and infiltrating macrophages. With recent literature suggesting that DSC1 inhibition promotes reduction in the atherosclerotic plaque, this mechanism is a promising new avenue for patients suffering from atherosclerosis and CAVS.

## Statement of Ethics

This study was approved by the Research Ethics Board of the McGill University Health Centre (Montreal, QC, Canada) with Approval No. 2014-1042: 13-025 GEN. Written informed consent was obtained from all participants prior to enrollment.

## Conflict of Interest Statement

The authors declare that they have no conflicts of interest, whether actual or perceived, related to this work.

## Funding Sources

K.K. is a recipient of the Canadian Institutes of Health Research Vanier Scholarship. This study was not supported by any sponsor or funder.

## Author Contributions

K.K. and Adel S. conceived the study. K.K. and B.Y. performed the experiments and data analysis. Adam S. performed data analysis. S.S.D. provided the human samples. K.K. drafted the initial manuscript. K.K., B.Y., S.A., Adam S., J.G., S.S.D., and Adel S. contributed to and approved the final revision.

## Data Availability

The data that support the findings of this study are not publicly available due to privacy reasons but are available from the corresponding author upon request.
